# Sexual Quality of Life-Female (SQoL-F): Cultural Adaptation and Validation of European Portuguese Version

**DOI:** 10.3390/healthcare10020255

**Published:** 2022-01-28

**Authors:** Margarida Sim-Sim, Vicki Aaberg, Sagrario Gómez-Cantarino, Hélia Dias, Ermelinda Caldeira, Irene Soto-Fernandez, Cinzia Gradellini

**Affiliations:** 1 Comprehensive Health Research Centre Integrated Researcher (CHRC), Nursing Department, University of Evora, Escola Superior de Enfermagem S. João de Deus. Largo Sr. da Pobreza, 7000-811 Evora, Portugal; msimsim@uevora.pt (M.S.-S.); ecaldeira@uevora.pt (E.C.); 2 School of Health Sciences, Seattle Pacific University, Seattle, WA 98119, USA; aaberv@spu.edu; 3 Faculty of Physiotherapy and Nursing of the Toledo Campus, University of Castilla-La Mancha (UCLM), 45071 Toledo, Spain; 4 Health Sciences Research Unit: Nursing (UICISA: E), Coimbra School of Nursing (ESEnfC), 3004-011 Coimbra, Portugal; 5 Research Group Nursing, Pain and Care (ENDOCU), University of Castilla-La Mancha (UCLM), Av. Carlos III s/n, 45071 Toledo, Spain; 6 Instituto Politecnico de Santarém, Escola Superior de Saúde, Cintesis Integrated Researcher, Qt.ª do Mergulhão—Sr.ª da Guia, 2005-075 Santarem, Portugal; helia.dias@essaude.ipsantarem.pt; 7 Department of Education, School Nurse, Public School, 8, 45005 Toledo, Spain; Irene.soto.due@hotmail.com; 8 EdSex Project, University of Modena and Reggio Emilia, Azienda Unità Sanitaria Locale-IRCCS of Reggio Emilia, 42122 Reggio Emilia, Italy; cinzia.gradellini@ausl.re.it

**Keywords:** validation studies, psychometrics, cultural adaptation, linguistic validation, nursing students

## Abstract

The Sexual Quality of Life-Female (SQoL-F) questionnaire was developed with qualitative data to assess the impact of sexual dysfunction in women. Objectives: the aim was to conduct a cross-cultural adaptation and psychometric analysis of a European Portuguese version of the Sexual Quality of Life-Female questionnaire. Methods: Methodological study of the processes of translation and cultural adaptation. This is a retrospective study in which nursing students participated. Data collection: Lime Survey platform in a convenience sample was carried out in two stages, the latter being re-testing data. The instrument analysed, presented as a latent variable, consisted of 18 items on a Likert scale. The study was approved by the Ethics Committee. Participants: the sample was 113 women, mean age 21.99 years (±3.76), attending classes in the first 4 years of the first cycle of nursing. Results: Reliability was analysed and stability was found in the test–retest (rs = 0.658) and in the intraclass coefficient (rs = 0.821). The internal consistency analysis showed an alpha value of 0.846. Discriminant validity analysis using the Mann–Whitney test revealed a higher score of the quality of sexual life of students living with parents/surrogates. Factor validity analysis was conducted using Oblimin rotation with four-, three- and two-factor tests. Parallel analysis of the empirical matrix compared to the random matrix showed that the instrument was unidimensional. Conclusions: the assessment of the properties of the SQoL-F is valuable, as the provision of a valid and reliable instrument contributes to the quality of subsequent studies, including for local and multicentre research.

## 1. Introduction

Sexuality is an inseparable part of humanity which reveals itself in the biological, psychological and social spheres and covers gender roles, sexual orientation, reproduction, and eroticism [1]. In Western culture female sexuality is steeped in stereotypes and stigmata which hinder the exploration and discussion of sexual life, seeking help or reflecting on quality of sexual life [2,3,4]. 

The concept of a balanced sexual life is associated with the idea that a satisfactory quality of sexual life is oriented toward a healthy lifestyle [5,6]. Satisfactory sexual life can occur in individuals of all ages, in positive and negative relationship contexts and with diverse life experiences [7,8]. Among youth and in the university environment, the quality of a young woman’s sexual life is above all viewed in terms of risks, quantity of partners and types of relationships, all of which are discussed without reliable research instruments for the evaluation of the quality of sexual life [8]. The quality of sexual life is made up of an individual’s understanding of the living out of one’s sexuality and includes sexual responses, sexual awareness and attitudes toward sexuality as well as reported experiences of intimate relationships and bodily and emotional sensations [9]. 

Examples of research tools for the assessment of the quality of sexual life include the PROMIS (Patient-Reported Outcomes Measurement Information System) Satisfaction with Sex Life questionnaire [10]. More comprehensive and rigorous study necessitates either the creation of original tools or the validation of existing ones. The SQoL-F is an 18-item instrument presented on a Likert scale scored from agree completely to disagree completely. The total score is obtained from the simple sum of items after reversing those that are formulated in a positive way (items 1, 5, 9, 13, 16 and 18). Higher scores reflect a higher quality of sexual life. The development of the several forms of this instrument involved interviews with 82 women from seven countries (Europe and U.S.). In the original study, the final scale revealed a high internal consistency with a Cronbach’s alpha coefficient of 0.950 [3]. The SQoL-F has proven useful among clinical populations including women with spinal cord injuries [11] and Polish women over 60 [12]. While several translations of the instrument have been validated [11,13], present research has revealed no Portuguese version for non-clinical populations. The aim of this study was to perform a linguistic–cultural adaptation and validation of a Portuguese version of the SQoL-F.

## 2. Materials and Methods

### 2.1. Study Design

A design validation test was used for the validation of the SQoL-F. 

### 2.2. Translation, Cultural Perspective of Semantics and Validation

**Figure 1:**
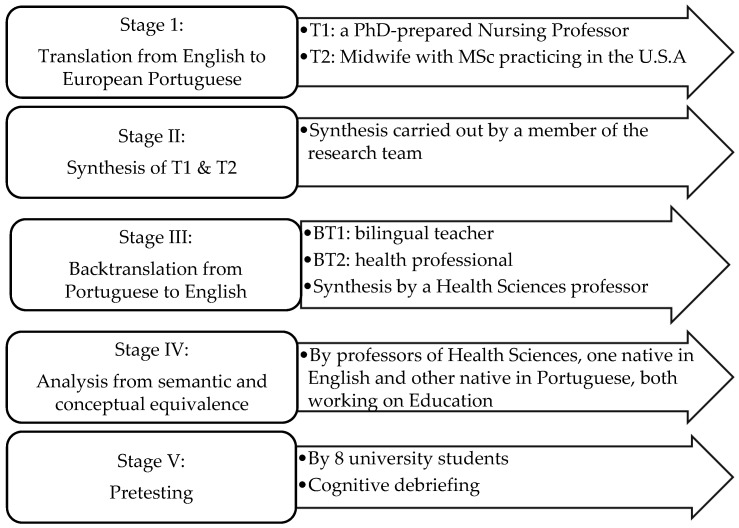
Stages of cross-cultural adaptation.

The author’s model [14] and the author’s recommendation [15] guided the process of adapting the SQoL-F for European Portuguese. There are 5 steps, ending with a cognitive debriefing [14,15], which revealed no difficulties or suggestions for changes. The steps are detailed in Figure 1.

### 2.3. Participants and Setting

The participants are university students recruited in a multicenter study on sexuality. The local representatives of the research team described the project in classrooms and invited students to participate. The sample size of a minimum of 90 students was arrived at, since the literature proposes 5–10 participants for each item in a research tool [16]. Inclusion criteria for the validation of this instrument were: attending the first cycle of nursing education, being female, having an affective-sexual relationship experience. Exclusion criteria were: (a) having children, which can be outliers in a sample of students; (b) being an international student, because the questionnaire is introduced in the Portuguese language; (c) being in a program for special needs students, because these conditions can introduce bias. 

### 2.4. Data Collection Procedure

Data was collected using the LimeSurvey platform with a convenience sample of 225 female students. Those who did not currently have a sexual partner (n = 66) or had never had a sexual partner (n = 26) were rejected. In addition, 20 who did not answer all of the SQoL-F items were excluded, and so 113 completed questionnaires were analyzed. Responses to the second application, carried out 3 to 6 weeks after the first, totaled 73 fully answered questionnaires.

As the questionnaire was applied by Lime Survey platform, the first question introduced informed consent. In a compulsory question, it was asked if students participated voluntarily. Only after students inserted “YES” on this compulsory question would it be possible to complete the questionnaire.

### 2.5. Data Analysis

A descriptive analysis focused on frequency and percentages, as well as measures of central tendency, was carried out. Distribution was analyzed using the Kolmogorov–Smirnov test, verifying non-normality (K-S = 0.254; df = 113; *p* < 0.001). The work of various authors guided the analysis of reliability and validity [15,17,18,19,20,21]. 

#### 2.5.1. Reliability

Reliability was measured through two criteria: (a) stability through the test–retest and (b) intraclass correlation coefficient (ICC) derived from the results of the second application. Internal consistency was measured via Cronbach’s alpha. 

For the ICC, based on a confidence interval of 95%, the following parameters were used: (a) weak if <0.50; (b) moderate, from 0.50 to 0.75; (c) good, from 0.75 to 0.90 and (d) excellent if >0.90. For internal consistency, the analysis employed: (a) unacceptable, if less than 0.50; (b) questionable, between 0.50 and 0.60; (c) acceptable, between 0.60 and 0.70; (d) good, between 0.70 and 0.80; (e) very good, between 0.80 and 0.90 and (f) excellent when greater than 0.90 [15,20,22]. 

#### 2.5.2. Validity

Determination of validity involves the observation of: (a) criterion validity; (b) construct validity, through convergent validity; (c) discriminant validity and (d) construct, structural or factorial validity [15,19,20]. 

For (a) criterion validity (or more precisely, for predictive validity), the observation is made through the correlation between the SQoL-F and a variable involved in overall sexual quality of life, presented in a 6-point scale (1 = very bad; 2 = bad; 3 = reasonable; 4 = good; 5 = very good; 6 = excellent) applied to the results of the second collection of data. This variable was designated by the acronym QL-Global. A correlation coefficient close to 0.600 or more is needed to demonstrate predictive validity [20,21]. 

(b) construct validity, through convergent validity, is observed in the relationship between the SQoL-F and the Attitudes and Beliefs Questionnaire (QACSES) (Carvalho et al., 2016). QACSES is a Portuguese instrument for the study of attitudes and beliefs related to sexuality. Positive construct validity is established with correlation coefficients of 0.600 or more. 

(c) construct validity, through discriminant validity, is observed through comparison of SQoL-F scores from university students living with their parents, those living in university residences or in rented rooms to scores from students living in a home with colleagues or non-parents.

(d) structural or factorial validity is observed through factorial analysis of principal components. Observation of the scree plot was performed using oblique rotation. The criteria for factor retention are: Eigenvalues > 1.0, items with factor loading > 0.30 and Pearson’s correction r > 0.30. 

(e) transcultural validity is established by evidence that attests similarities between the current study and others that use SQoL-F in a similar population. 

IBM SPSS^®^ software, version 26, was used. A significance level of 0.05 was assumed.

### 2.6. Ethical Procedures

The study formed part of a multicenter research project with approval from the Ethics Committee for Research in the Areas of Human Health and Welfare of the University of Évora (Registration No. 18175). Out of respect for intellectual property, permission was sought from the original authors of SQoL-F. Permission was granted via email on 1 July 2019. The original authors were cited. 

## 3. Results

### 3.1. Characteristics of Participants

The 113 participants were between 18 and 40 years old (M = 21.99; DP = 3.760), with a median age of 21 and mode of 22. Using the “Visual Binning” feature in SPSS, age was categorized as a percentage according to the digitized surveys with two cutoff points, with the group of youngest students being the most represented. The students were enrolled in 1 of 4 years of the first cycle of nursing education, with first-year students the most represented (n = 34; 30.1%). Using the chi-squared test of adherence, it was observed that the real frequencies conformed to the frequencies predicted in the age groups (*p* = 0.202) and the curricular year groups (*p* = 0.571). Differences were found during consideration of living situations, with cohabitation with parental figures the most represented. Most participants reporting having a sexual partner (n = 111; 98.2%), with only two participants reporting having multiple partners (1.8%) (Table 1). 

### 3.2. Reliability

Stability through the test –retest was established by the second application of the SQoL-F (T2 SQoL-F). In total, 73 students answered all questions and the variable T2SQoL-F emerged. A positive association was observed between SQoL-F and T2SQoL-F (rs = 0.658; n = 73; *p* < 0.001). Regarding the ICC, between SQoL-F and T2SQoL-F, a coefficient of 0.821 was obtained. The internal consistency of the SQoL-F was demonstrated by a Cronbach’s alpha coefficient of 0.846. The items show an item-total correlation that varies between −0.059 (item 16) and 0.878 (item 3) (Table 2).

The description of the SQoL-F items, in the original and in the Portuguese version, can be found in the Table 3.

### 3.3. Construct Validity

The instrument was completed in 8–10 min, suggesting good face and content validity.

Predictive validity, in the association of SQoL-F compared to QL-Global, revealed a positive correlation (n = 73; rs = 0.605 *p* < 0.001). In the analysis of convergent validity, the association between SQoL-F compared to QACSES, revealed positive and significant values (n = 113; rs = 0.338; *p* < 0.001).

**Figure 2:**
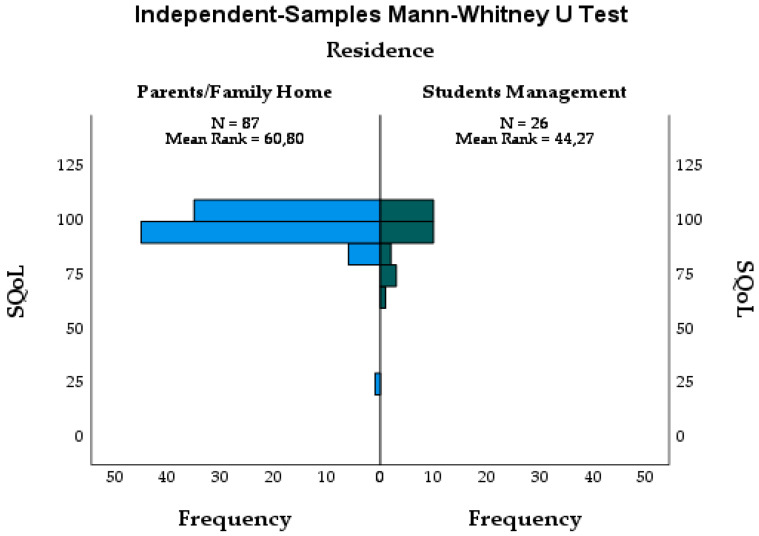
Mann–Whitney test. Sources: authors’ own elaboration.

Analysis for discriminant validity through a Mann–Whitney test revealed that the group of university students who live in parental residence or managed by parental substitutes have a higher average rank (n = 87; Mean Rank = 60.80) than university students living with colleagues or another non-parents (n = 26; Mean Rank = 44.27), with significant differences (U = 800.000; Z = −2.267; *p* = 0.023) (Figure 2).

Structural or factorial validity was observed through principal components analysis (PCA). With the Kaiser–Meier–Olkin coefficient of 0.857 and Bartlett’s Sphericity Test （$\chi_{\left(153\right)}^{2}$ = 160.407; *p* < 0.001), the sample was shown to be adequate for factor analysis. Oblique rotation (Oblimin with Kaiser normalization) was performed, and the initial solution showed four factors. The third factor included only one item (item 10). The total variance explained with Eigenvalues above 1 is 71.11%, with the first factor being 48.16%. A new analysis was performed on three factors. Altogether they explain 64.88% of the variance. All items were saturated above 0.535 (Table 4).

As the slope diagram suggested a unidimensional construct, parallel analysis was employed to compare the empirical matrix to the random matrix. Two factors emerged, whose magnitude of variance was higher in the empirical matrix (Table 5). 

**Figure 3:**
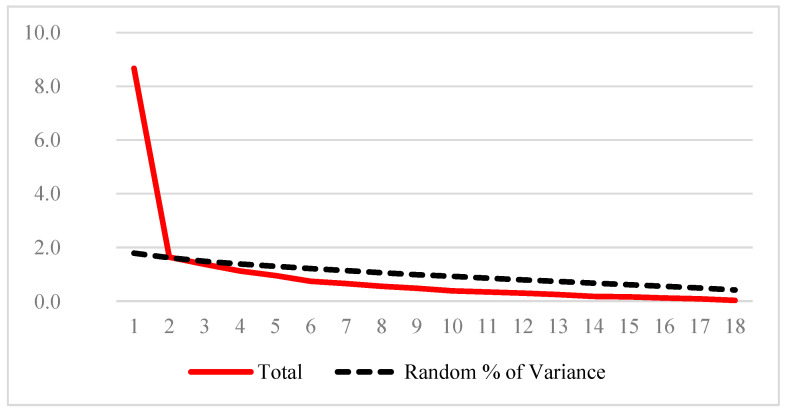
Variance parallel analysis of empirical and randomized factors.

Syntax was used. The graphical representation of the empirical matrix solution versus the random matrix solution shows the cutoff point of the two lines, suggesting unidimensionality (Figure 3).

The average in the SQoL-F was 96.115 (SD = 9.45), with a minimum of 25 and a maximum of 105. Considering the cutoff points used in a previous study [11], the participants’ quality of life is the following: one participant (0.9%) considers that she has a low SQoL-F, eight (7.1%) consider their SQoL-F to be moderate and the majority (n = 104; 92%) report that their SQoL-F is high. 

## 4. Discussion

The participants have characteristics similar to participants in other studies where SQoL-F was applied and validated in other languages [23]. The low number of responses may be attributable to the collection of data by digital means. Although digital data collection offers the advantage of greater adaptability to personal schedules, the request for collaboration seems to have been ignored often, despite reminders. Digital response rates have been decreasing since the 1980s and are generally low [24]. However, the sample size is in agreement with some authors, who have used the same norm to validate the SQoL-F [23]. Research has shown that the current study is the first in the country to carry out the cross-cultural adaptation and a psychometric study of the SQoL-F for European Portuguese.

Regarding cross-cultural adaptation, the Beaton et al. model serves as authoritative guide for the validation of instruments applied to nursing [25]. Interpretation of the survey by participants demands strict adherence to the steps outlined in this model. In the current study, five steps were carried out. Beaton, et al. (2000) suggest a sixth step, which was not possible to perform in this instance.

In Portugal, although there are publications that analyze research tools for the health sciences [26], there is no institution for the registration of culturally adapted or original versions. In the absence of formal registration and the absence of consensus on the need for permission from the original author [27], copyright was respected by direct request and guarantee of citation. When it is desired to validate or replicate an instrument, although there is a prospect that a published article is public knowledge [26,27], the request for permission and the citation respect intellectual property.

### 4.1. Reliability

Regarding the evaluation of the stability of the SQoL-F, the number of participants in this study exceeded the minimum number (more than 30 cases) for application of the intraclass correlation coefficient (ICC) [22]. The ICC value (0.821) was satisfactory [3,22]. The result is similar to those of other studies that found coefficients of 0.88 [23] and 0.85 [3].

In the analysis of internal consistency of SQoL-F, the Cronbach’s alpha coefficient showed good values (>0.800). The alpha coefficient shows the level of agreement of the manifest variables and is greatly influenced by the number of these variables, since a small number lowers the alpha scores [21]. The item-total correlations were satisfactory for most items. Being high, they show that the set of items measure the same construct, attesting to reliability [15] (also see Souza [21]). The decision was made to keep the two items with a lower item-total correlation, since their removal would not cause a major increase in the value of Cronbach’s alpha. In the current sample, the internal consistency values are in agreement with those of other studies of the SQoL-F [3,11,23]. 

### 4.2. Validity

Analysis for predictive validity, through comparison between SQoL-F and the criterion measure (QL-Global) implemented later, revealed satisfactory values. This suggests, therefore, that in a global understanding of sexual life (QL-Global) a positive association with SQoL-F is foreseeable. In the studies consulted, no analysis of predictive validity was identified. The results contribute to theoretical considerations about this parameter, since the current coefficient value (0.556), is close to 0.600 [23,28]. The performance forecast of SQoL-F appears to be adequate.

Convergent validity, examined in the relationship between SQoL-F and QACSES [29], approached expectations, since the comparison presented a significant Spearman Rho Coefficient. However, the correlation value is low compared to the standard set by some authors. The studies by Symonds [3] show correlations between 0.500 and 0.700 in one sample and 0.480 and 0.332 in another sample. Thus, the current value 0.338 in Spearman’s Rho does not seem to be absolutely inappropriate. Perhaps the QACSES may not be the ideal parameter for testing convergent validity, since the instrument is multidimensional.

Analysis of discriminant validity through the Mann–Whitney test brought controversial results. The expectation was that the highest SQoL-F rakings would be observed in girls living in accommodation with colleagues, as less parental/substitute control leads to more affective-sexual experiences, greater personal freedom, and greater satisfaction with sexual life [29]. The lower SQoL-F scores observed in women who live with colleagues suggests that there will be some affective-sexual fragility, a situation experienced by pregnant and postpartum women in a Spanish study [30]. On the other hand, the results are consistent with an American study in which female university students reported negative feelings about their sex lives [31,32]. During university studies, individuals have sexual experiences which satisfy curiosity and form sexual identity and relationships. In this phase of life, relationships emerge which, due to emotional immaturity or a desire to experiment, do not tend to be long-lasting. 

Structural or factorial validity was explored from several perspectives. While the original study had a unidimensional construct [3], validation for the Iranian population pointed to four dimensions [23] which are also recognized in women with spinal cord disease [11]. On the other hand, a Kenyan study has observed SQoL-F from a unidimensional perspective [33,34].

The option for Oblimin oblique rotation was linked to the perspective of the original author, who supports the idea of related underlying factors [3,18]. Oblimin rotation was used due to the correlation between factors [35]. Although most factor analyses are carried out with Varimax rotation, for the current case, Oblimin rotation supported the data better since it did not limit the analysis to being orthogonal. 

The first and second AFCP showed factor saturation greater than 0.400, but the organization of the items was not intelligible. In the screeplot observation, the existence of an inflection point of the curve, contributing to the separation of principal factors and trivial factors [18], suggested a unidimensional construct. However, as the analysis of the slope diagram is more robust in samples with more than 200 cases [18], the move was made to parallel analysis, suggesting in the graph the unidimensionality of the instrument. In a conservative approach, it was decided to understand the construct as unidimensional. This is in line with the original study, where the intention was to move toward a unidimensional construct [3,36]. 

## 5. Conclusions

Measurement validation studies are useful because making instruments available in a language other than the original provides the basis for higher-quality results and expanded possibilities for future local and multicenter research. The systematization of the translation-back-translation and colloquial adaptation processes was fundamental, since the variables are only comprehensible when the message is assimilable and conveys the content. The analysis of reliability through the ICC and internal consistency confirmed the capacity of the instrument to reproduce stable and accurate results. The SQoL-F has now been reliably replicated in European Portuguese since the reliability index has been demonstrated by the ratio of the true variance to the true variance plus the error variance. In studies using instruments originally developed in another language and/or context, the information collected and the quality of the results are influenced by the rigor of the validation process.

The non-random sample prevents generalization of the results. The second data collection did not fully cover the time span of 2 to 4 weeks. The stages for cross-cultural adaptation are inspired by a mixed model. The number of people who have participated is reduced. The instrument performed well but it will be useful to test convergent validity with another scale. More studies are needed, with a larger and randomized sample, so that the unidimensionality may be confirmed or refuted. 

## Figures and Tables

**Table 1:** Participant Characteristics.

Characteristics		Participants n (%)	Chi-Squared
Age	≤20 years	44 (38.9)	$\chi_{\left(2,113\right)}^{2}$ = 3.204; *p* = 0.202
	21–22 years	40 (35.4)	
	>23 years	29 (25.7)	
Year of Study	1st year	34 (30.1)	$\chi_{\left(3,113\right)}^{2}$ = 2.009; *p* = 0.571
	2nd year	29 (25.7)	
	3rd year	24 (21.2)	
	4th year	26 (23.0)	
Residence	Parents house	52 (46.0)	$\chi_{\left(4,113\right)}^{2}$ = 58.106; *p* < 0.001
	University housing	8 (7.1)	
	Rented room	27 (23.9)	
	Rented house with roommates	9 (8.0)	
	Other	17 (15.0)	
Total		113 (100)	-

Sources: authors’ own elaboration.

**Table 2:** Descriptive statistics and internal consistency for the items.

Item	Mean	Std. Deviation	Corrected Item-Total Correlation	Cronbach’s Alpha If Item Deleted
* SQoL-F1	5.62	0.672	0.716	0.831
SQoL-F2	5.72	0.785	0.748	0.828
SQoL-F3	5.83	0.625	0.878	0.827
SQoL-F4	5.82	0.697	0.838	0.826
* SQoL-F5	5.49	0.888	0.672	0.829
SQoL-F6	5.78	0.765	0.684	0.831
SQoL-F7	5.39	1.097	0.534	0.834
SQoL-F8	5.89	0.541	0.835	0.831
* SQoL-F9	5.34	0.786	0.658	0.831
SQoL-10	2.68	1.670	−0.154	0.887
SQoL-F11	5.84	0.591	0.617	0.836
SQoL-F12	5.51	0.974	0.493	0.836
* SQoL-F13	5.62	0.899	0.747	0.826
SQoL-F14	5.63	1.028	0.416	0.840
SQoL-F15	5.73	0.845	0.628	0.831
* SQoL-F16	3.46	1.866	−0.059	0.888
SQoL-F17	5.81	0.774	0.646	0.832
* SQoL-F18	4.94	1.325	0.434	0.841

Sources: authors’ own elaboration * reversed items.

**Table 3:** Item description of the SQoL-F Questionnaire.

Item	English Description and Portuguese Description
* SQoL-F1	1. When I think about my sex life I feel, I think that generally it is a pleasant part of my life * 1. Quando penso na minha vida sexual, acho que em geral, é uma parte agradável da minha vida *
SQoL-F2	2. When I think about my sex life I feel frustrated 2. Quando penso na minha vida sexual, sinto-me frustrada
SQoL-F3	3. When I think about my sex life I feel depressed 3. Quando penso na minha vida sexual, sinto-me deprimida
SQoL-F4	4. When I think about my sex life I feel less of a woman 4. Quando penso na minha vida sexual, sinto-me menos mulher.
* SQoL-F5	5. When I think about my sex life I feel good with myself * 5. Quando penso na minha vida sexual, sinto bem comigo mesma *
SQoL-F6	6. I lost confidence in myself as a sexual partner 6. Perdi a confiança em mim como parceira sexual.
SQoL-F7	7. When I think about my sex life I feel anxious 7. Quando penso sobre a minha vida sexual, sinto-me ansiosa.
SQoL-F8	8. When I think about my sex life I feel rage 8. Quando penso sobre a minha vida sexual, sinto raiva
* SQoL-F9	9. When I think about my sex life I feel closer to my partner * 9. Quando penso na minha vida sexual, sinto-me mais próxima do meu parceiro *
SQoL-10	10. I worry about the future of my sex life 10. Preocupo-me com o futuro da minha vida sexual
SQoL-F11	11. I lost pleasure in sexual intercourse 11. Perdi o prazer na atividade sexual
SQoL-F12	12. When I think about my sex life I feel embarrassed 12. Quando penso na minha vida sexual, fico embaraçada
* SQoL-F13	13. When I think about my sex life I feel I can speak to my partner about sexual matters * 13. Quando penso acerca da minha vida sexual, sinto que posso falar com o meu parceiro sobre questões de índole sexual *
SQoL-F14	14. I try to avoid sexual intercourse 14. Eu tento evitar ter atividade sexual
SQoL-F15	15. When I think about my sex life I feel guilty 15. Quando penso acerca da minha vida sexual, sinto-me culpada
* SQoL-F16	16. When I think about my sex life I am scared that my partner will feel hurt or rejected * 16. Quando eu penso na minha vida sexual, preocupo-me se o meu parceiro se sente magoado ou rejeitado *
SQoL-F17	17. When I think about my sex life I feel as if I have lost something 17. Quando penso na minha sexual, sinto-me como que tenha perdido algo
* SQoL-F18	18. When I think about my sex life I am satisfied the frequency of sexual intercourse * 18. Quando eu penso sobre a minha vida sexual, estou satisfeita com a frequência da atividade sexual *

* Reversed items.

**Table 4:** Exploratory factorial analysis considering the principal components method with Direct Oblimin rotation (with Kaiser normalization) for four and three factors.

	Component				Component		
	1	2	3	4	1	2	3
SQoL3F	0.945			−0.551	0.883	0.555	
SQoL4F	0.926			−0.460	0.846	0.530	
SQoL8F	0.892			−0.570	0.831	0.605	
SQoL17F	0.802				0.676	0.477	
SQoL6F	0.789		−0.406	−0.447	0.614	0.758	
SQoL15F	0.767			−0.460	0.556	0.806	
SQoL2F	0.757			−0.561	0.804	0.404	
SQoL13F	−0.737			0.554	−0.785		
SQoL1F	−0.673			0.629	−0.774	−0.410	
SQoL11F	0.669		−0.491	−0.578	0.576	0.727	
SQoL14F	0.637		−0.598			0.884	
SQoL5F	−0.631	0.483		0.534	−0.768		
SQoL16F		0.704					0.727
SQoL18F		0.544		0.454	−0.620		
SQoL10F			0.856				0.708
SQoL7F	0.448			−0.825	0.603		
SQoL12F	0.437			−0.801	0.535		
SQoL9F	−0.509	0.552		0.676	−0.760		

Extraction Method: Principal component analysis. Rotation Method: Oblimin with Kaiser Normalization.

**Table 5:** Magnitude of variance of the empirical and randomized matrices.

	Matrix	
	Empirical	Randomized
1	8.669	1.788
2	1.638	1.613
3	1.371	1.482
4	1.121	1.382
5	0.953	1.296
6	0.740	1.212
7	0.655	1.138
8	0.552	1.059
9	0.478	0.985
10	0.381	0.923
11	0.338	0.855
12	0.296	0.793
13	0.242	0.731
14	0.175	0.667
15	0.157	0.613
16	0.118	0.552
17	0.085	0.489
18	0.029	0.417
Total	18.00	18.00

Sources: authors’ own elaboration.
